# Carbon budgets of potential tropical perennial grass cropping scenarios for bioenergy feedstock production

**DOI:** 10.1186/s13021-018-0102-8

**Published:** 2018-09-24

**Authors:** Meghan Pawlowski, Manyowa N. Meki, James R. Kiniry, Susan E. Crow

**Affiliations:** 1 Dept. of Natural Resources and Environmental Management, University of Hawaii at Manoa, 1910 East-West Rd., Honolulu, HI 96822 USA; 2 Texas A & M AgriLife Research, Blackland Research and Extension Center, 720 E. Blackland Rd., Temple, TX 76502 USA; 3 USDA, Agricultural Research Service, Grassland Soil and Water Research Laboratory, 808 E. Blackland Rd., Temple, TX 76502 USA

**Keywords:** Global warming potential, Greenhouse gas index, Napiergrass, Ratoon crop, Sugarcane

## Abstract

**Background:**

The environmental costs of fossil fuel consumption are globally recognized, opening many pathways for the development of regional portfolio solutions for sustainable replacement fuel and energy options. The purpose of this study was to create a baseline carbon (C) budget of a conventionally managed sugarcane (*Saccharum officinarum*) production system on Maui, Hawaii, and compare it to three different future energy cropping scenarios: (1) conventional sugarcane with a 50% deficit irrigation (sugarcane 50%), (2) ratoon harvested napiergrass (*Pennisetum purpureum* Schumach.) with 100% irrigation (napier 100%), and (3) ratoon harvested napiergrass with a 50% deficit irrigation (napier 50%).

**Results:**

The differences among cropping scenarios for the fossil fuel-based emissions associated with agricultural inputs and field operations were small compared to the differences associated with pre-harvest burn emissions and soil C stock under ratoon harvest and zero-tillage management. Burn emissions were nearly 2000 kg C_eq_ ha^−1^ year^−1^ in the conventional sugarcane; whereas soil C gains were approximately 4500 kg C_eq_ ha^−1^ year^−1^ in the surface layer of the soil profile for napiergrass. Further, gains in deep soil profile C were nearly three times greater than in the surface layer. Therefore, net global warming potential was greatest for conventional sugarcane and least for napier 50% when deep profile soil C was included. Per unit of biomass yield, the most greenhouse gas (GHG) intensive scenario was sugarcane 50% with a GHG Index (GHGI, positive values imply a climate impact, so a more negative value is preferable for climate change mitigation) of 0.11 and the least intensive was napiergrass 50% when a deep soil profile was included (GHGI = − 0.77).

**Conclusion:**

Future scenarios for energy or fuel production on former sugarcane land across the Pacific Basin or other volcanic islands should concentrate on ratoon-harvested crops that maintain yields under zero-tillage management for long intervals between kill harvest and reduce costs of field operations and agricultural input requirements. For napiergrass on Maui and elsewhere, deficit irrigation maximized climate change mitigation of the system and reduced water use should be part of planning a sustainable, diversified agricultural landscape.

## Background

Interest in the production of renewable biofuel from lignocellulosic crops is gaining global recognition as a leading alternative energy scenario in future fuel markets. The negative environmental costs associated with fossil fuel consumption are being recognized and accounted on an international level due primarily to the adoption of the Kyoto Protocol in 2005. Since then, the development of alternative fuel sources has been a major concern for nations like the United States that rely heavily on imported fossil fuel. These pressures can result in a shift from the conventional food-crop agriculture to bioenergy systems in the United States and production of renewable biomass sources has been accelerated in recent years due to government regulations [1]. More specifically, regulations such as the Energy Independence and Security Act (EISA) of 2007 have mandated that fossil fuel sources must be mixed with at least 36 billion gallons of renewable fuel by the year 2022, meeting approximately 25% of liquid-based fuel needs by 2050 [2]. In support of national policy, Hawaii is under similar pressure resulting from the 2008 Hawaii Clean Energy Initiative (HCEI), which mandates a local, efficient, and renewable source of fuel be developed for Hawaii by the year 2030. The HCEI specifically requires that 40% of the states fossil energy be replaced with “locally generated renewable sources” over the next 17 years. These increasing fuel demands may place additional stress on already exploited agricultural lands resulting in land intensification and conversion if not managed conservatively.

Agricultural intensification can negatively impact soil carbon (C) storage, increase greenhouse gas (GHG) emissions, and offset the overall ecosystem C balance if managed incorrectly [2–4]. The energy sector en masse converted annual croplands into perennial biomass systems over the past decade, which was thought to provide GHG mitigation potential through an improvement in soil quality and a reduction in nutrient amendments [5, 6]. In recent years, however, conflicting results about the mitigation potential of these systems raised questions about the true system-level GHG offset [3, 7, 8]. Shortages in the world’s productive croplands and the impending scarcity of water made worse by climate change lead to additional uncertainty in the sustainability of increasing the world’s biofuel acreage [9, 10]. Due to the wide range of conflicting viewpoints on the bioenergy issue, documentation of local and regional datasets that quantify these uncertainties are of the utmost importance.

The total GHG balance of biofuel production is difficult to capture because of the large variation in cropping practices, land management, and equipment usage across these agricultural systems. Additionally, many recent studies focused on single-species scenarios that are specific to Brazilian ethanol production and are not entirely applicable to addressing regional issues outside of their study areas [3, 8, 11, 12]. Although these studies can be a powerful tool to advance the global implications of bioenergy production under a large agricultural infrastructure, additional small-scale studies are required to assess the tradeoffs between these systems and provide alternative management options on a local scale. To do this, a GHG balance needs to be created based on site-specific data that incorporates the energy inputs (fossil and non-fossil fuel) required to establish and maintain a bioenergy cropping system. For the majority of these GHG assessments, fossil energy inputs are converted using emission factors to carbon dioxide equivalents (CO_2eq_). Referred to as a global warming potential (GWP), these CO_2eq_ are comparable on an international scale. This kind of GHG accounting allows for a direct comparison between the GHGs emitted by agricultural operations and the GHGs saved by the production of a renewable fuel sources [3, 8]. For the purposes of GHG accounting, all emissions were converted into a similar C equivalent (C_eq_) in order to make a direct comparison with the amount of soil C stored under each cropping practice. This C budget related the GWP of each of these systems to their crop production, which allows for estimation of the overall GHG intensity (GHGI) of a production system [13].

To date, there has been no known C budget specific to the agricultural operations in Hawaii, which makes comparing future cropping scenarios against current practices impossible. With over 11,000 ha^−1^ of Maui being state designated and protected agricultural land, the future sustainability of this region may lie with the adoption of an energy crop scenario that is able to displace fossil fuel based GHG emissions. The purpose of this study was to create a baseline C budget for a conventionally managed sugarcane production system on (100% irrigation) Maui and compare it to three different future energy cropping scenarios: [1] conventional sugarcane with a 50% deficit irrigation (sugarcane 50%), [2] ratoon harvested napiergrass with 100% irrigation (napier 100%), and [3] ratoon harvested napiergrass with a 50% deficit irrigation (napier 50%). This comparison will help identify, in terms of their agricultural C budgets, the best-case scenario for future biofuel production in Hawaii and other Pacific Basin island nations.

## Methods

### Site description and experimental design

The field experiment was conducted in central Maui, Hawaii (20.89°N, 156.41°W) on Hawaiian Commercial and Sugar Company (HC&S) land. At the time of the study, HC&S was the only remaining sugarcane plantation in Hawaii. The experimental plots were within a highly weathered, very-fine, kaolinitic, isohyperthermic Typic Eutrotorrox of the Molokai series in field #609, which is approximately 100 meters above sea level and has a total commercial area of 72 ha. The soil is well drained, rocky, and has deep, well-defined horizons below the plow layer [14]. Soil pH was 7.97, and C concentration was 1.37% on average in the top 40 cm with a mean bulk density of 1.51 g cm^3^ as assessed by the baseline soil collection in 2011. During the trial period, average annual air temperature was 23.4 °C and annual precipitation was 241 mm, which are consistent with long-term averages for the area [15].

The experiment was a strip-plot, group-balanced design with two factors, irrigation and species with three replicates (blocks) (please see [16] for additional details). Irrigation was applied at the standard plantation rate (100%), and two deficit irrigation rates (75% and 50% of plantation standard). The original trial included four species, sugarcane (*Saccharum officinarum*), energycane (*Saccharum officinarum x Saccharum spontaneum*), napiergrass (*Pennisetum purpureum* Schumach.), and sweet sorghum (*Sorghum bicolor* (L.) Moench). For this study, two crops (sugarcane and napiergrass) were evaluated at two irrigation levels (50% and 100%). On June 26, 2011 the field plots were established in a recently harvested sugarcane field that had been in a cane-on-cane rotation for over 100 years. The sugarcane plots were planted with seed cane from an adjacent field within the HC&S plantation and napiergrass seed crop was supplied from a harvested population at the University of Hawaii’s research station in Waimanalo, Oahu.

Deficit irrigation treatments were applied to the field from November 13, 2011 depending on water availability across the plantation and were controlled with automated timers. From November 2011–October 2012, 1245 mm water ha^−1^ were applied to the 100% plots and 633 mm water ha^−1^ were applied to the 50% plots, for an actual deficit treatment of 50.8%. During the study period, the napiergrass plots were harvested every 6 months (a time interval that maximizes yield) on March 13, 2012 and September 25, 2012. The sugarcane crop was under a 2-year growth rotation and did not reach maturity during the scope of this study period and was not harvested.

### Developing a baseline system for sugarcane

Sugarcane is a high-yielding, tropical C4 perennial grass of South Pacific origin. Tropical sugarcane biomass yields are up to 40 Mg dry wt ha^−1^ year^−1^ in Hawaii and 26 Mg dry wt ha^−1^ year^−1^ in Brazil [8, 17]. The species supports a drought resistant, robust root system that can improve soil structure and accumulate C on marginal lands [18, 19]. However, Hawaiian sugarcane has been grown on a 2-year crop cycle, reaching maturity after 24 months and then harvested after a low intensity burn followed by deep tillage and mechanized planting. Commercial sugarcane production existed on Maui for over 125 years. However, in January 2016 HC&S, the last remaining large-scale sugar producer, announced a wholesale transition on their 14,000 ha plantation to diversified agriculture including perennial grasses for forage, pasture, and bioenergy feedstock.

The development of a C budget or GHG analysis includes a detailed accounting of the agricultural inputs required to produce a specific crop. This includes quantifying the fossil fuel and non-fossil fuel based emissions. Fossil emissions are considered emissions resulting from fuel use during field preparation, planting, application of agrochemicals, harvesting, and maintenance [3, 5, 11, 12, 20]. Non-fossil emissions are considered biogenic GHG emissions that consider the production of carbon dioxide (CO_2_), methane (CH_4_), and nitrous oxide (N_2_O) as a result of the production system and are primarily a result of pre-harvest burn operations, soil GHG exchange, and residue management [3]. This baseline system will be referred to as the sugarcane 100% scenario for future comparisons.

### Fossil emissions from fuel consumption and agricultural inputs

#### Field operations

Field operations (i.e., field preparation, harvest, fabrication and maintenance, seed propagation, and irrigation) often are considered to be hidden sources of emissions because of their indirect contribution to GHG flux. These emissions are caused by the burning of fossil fuel during equipment operation [12] and accounting can be challenging partly because of large variation in the descriptive energy units [20]. A standard unit of kg C_eq_ was used to assess the contribution of field operations to the total C budget. Emissions factors (EF) were used to convert the fuel use requirements of each operation to C_eq_. The EFs used in this analysis represent a synthesis of the best-available and current values found in the literature. Specific information pertaining to type of equipment and usage has been obtained from personal communication with plantation staff.

When EFs found in the literature were inadequate to describe the operation on Maui, they were generated independently to best reflect current plantation practices. For example, irrigation emissions were calculated based on the average energy required to pump water across the plantation at a rate specific to Maui, 0.0057 L/kWh (personal communication with L. Jakeway, Chemical Engineer at HC&S, 2013). Additionally, this value was adjusted to account for the total amount of water applied to a field in the 2011–2012 year to correctly reflect the amount of renewable energy used on the plantation for these operations (approximately 3:1 renewable to fossil energy ratio). An additional input to the baseline scenario was a calculation of fossil emissions related to seed cane production, which were adjusted for the weight of seed cane used in planting operations [21].

#### Agricultural inputs

Emissions from agricultural inputs (i.e., fertilizer, herbicide, and lime) are a result of the energy required to produce, transport, and distribute these items [11, 20, 22]. A pre-emergence herbicide mix containing atrazine (1-chloro-3-ethylamino-5-isopropylamino-2, 4, 6-triazine), 2, 4-D (2, 4-dichlorophenoxyacetic acid), Prowl ((*N*-1- ethylpropyl)-3, 4-dimethyl-2, 6 dinitrobenzenamine), Rifle (3, 6-dichloro-2-methoxybenzoic acid), and Velpar (3-cyclohexyl- 6-dimethylamino-1-methyl-1, 3, 5-triazine-2, 4(1H,3H)-dione) was applied once three weeks after planting. Each plot received 345 kg N ha^−1^ (as liquid urea: 46-0-0) applied through the drip irrigation system. The fertilizer was applied monthly once the crops were established and concluded after 10 months. The timing and rate of urea application were optimized for the 2-year sugarcane crop and were based on current HC&S plantation practices. Deficit irrigation treatments were postponed during all fertilizer application events.

Quantification of the C emissions resulting from agricultural inputs in Maui sugarcane production was based on application rate and converted to C_eq_ with reported EF [11, 20]. An EF of 0.97 was used to convert the fertilizer application rate to C_eq_. Lime (CaCO_3_) was applied at a rate of 2569 kg ha^−1^ prior to field planting and converted to C_eq_ using an EF of 0.12. Individual emission factors were identified for each chemical used in the herbicide mix reported by HC&S. These factors were averaged and a new EF (5.64) was developed for herbicide application specific to Maui.

### Non-fossil emissions

#### Litter decomposition

Emissions from litter decomposition is a function of residue management. For example, N_2_O emissions can increase due to decomposition of leaf material following harvest and are greater in intact compared to burned fields [3]. The goal on Maui sugarcane fields has been to maintain 15% of total field biomass for crop residue. Using this percentage, litter C_eq_ were calculated based on an average biomass production of 80.4 Mg ha^−1^ year^−1^ reported by HC&S. Emission factors for litter decomposition are based on the amount of N in crop residues following harvest [3, 11, 12].

#### Pre-harvest burn emissions

Conventional cultivation of sugarcane in Hawaii included a pre-harvest, low intensity burn to remove unwanted leafy material prior to harvesting. Pre-harvest burning significantly increases GHG emissions through the production of CH_4_ and N_2_O and the release of black carbon (BC) to the environment [3, 12]. The values used for the cropping scenarios in Maui were based on a 15% residue retention rate of total field biomass. Current IPCC values outlined in the Guidelines for National Greenhouse Gas Inventories assessment in 2006 suggest an EF of 0.07 kg N_2_O per ton of dry matter burnt and an EF of 2.7 kg CH_4_ per ton of dry matter burnt. Black C has a GWP that is 500 times greater than CO_2_ on a 100-year time horizon and it is estimated that 1 kg of BC is created for every kg of trash burnt [12, 23].

#### Soil N_2_O and CH_4_ emissions

Field measurement of soil fluxes in CH_4_, and N_2_O began in October of 2011 and were sampled at least monthly until October of 2012 following the GRACEnet sampling protocols [24], as previously reported in detail (Pawlowski et al. in review). Mid-morning measurements were collected from sealed static PVC chambers affixed to permanent collars installed in the sugarcane and napiergrass rows and inter-rows. Samples were collected by sealing each chamber and using a 10 mL polypropylene syringe and extracting 8 mL of headspace air through a septum on the styrene lid at 0, 15, 30, 45, and 60 min after chamber closure. Each gas sample was immediately injected into an evacuated Exetainer^®^ (Labco Limited, UK) fitted with a Doubled Wadded Teflon/Silicon septa (Labco Limited, UK) for short-term storage. Samples were analyzed using a Shimadzu GC-2014 Gas Chromatograph (Shimadzu Scientific Instruments, Inc.), which used a flame ionization detector to measure the concentration of CH_4_ and CO_2_ after methanization, and an electrical conductivity detector for N_2_O analysis. Flux rates were calculated by assuming a linear change in gas concentration over time [25, 26]. Cumulative annual emissions of N_2_O and CH_4_ were interpolated from daily fluxes and summer over the first year and reported in terms of kg CH_4_ ha^−1^ year^−1^ and kg N_2_O ha^−1^ year^−1^ [2, 13]. To discuss N_2_O and CH_4_ emissions in terms of a C balance, annual rates were converted into CO_2eq_ using the IPCC 100-year horizon factors for calculating GWP. Therefore, when CO_2_ = 1 on a 100 year^−1^ time scale, then the GWP for N_2_O and CH_4_ are 298 and 25 respectively [26–28]. For the purposes of making direct comparisons with soil C storage on these plots, the GWP values were converted to C_eq_ relative to C in CO_2_.

### Soil C quantification

Ten baseline soil cores were collected in June 2011 using 20-cm depth increments up to a vertical depth of 2.4 m. Cores were extracted using a standard wet core diamond tipped drill bit with an internal diameter of 7 cm (Diamond Products Core Borer, Elyria, Ohio, USA). Each core barrel was inserted into the soil by a rotating hydraulic drill to minimize compaction within the barrel and to ensure accurate depth measurements. Soil samples were frozen at field moisture conditions until laboratory analysis. The cores were sieved at < 2 mm and dried for 48 h at 105 °C. Subsamples were ground to pass through a 250 micron sieve for heterogeneity, weighed, and analyzed for C concentration by combustion using a Costech ECS 4010 CNH Analyzer (Costech Analytical Technologies, Inc., Valencia, CA, USA). Soil C stock was determined with the equivalent soil mass method in increments of 3600 Mg ha^−1^ and a mean value for the baseline cores was determined at the 7200 and 18,000 Mg ha^−1^ reference masses, which represent the surface layer (25–40 cm) and deep profile (1–1.4 m) of soil [29].

Three soil cores (65-mm inner diameter bucket auger) were collected annually to a depth of 120 cm in 20 cm increments from each of the experimental plots. Samples were processed as noted above for the baseline cores for C concentration and soil C stock determination at the 18,000 Mg ha^−1^ reference mass. The change (∆) in soil C stock was made for each plot by subtracting the mean baseline value from the mean of the three cores for each plot and was previously reported [16]. The mean ∆ values for experimental years 1 and 2 were reported here as an annualized ∆.

### Alternative-cropping scenarios

With local and national mandates for cellulosic energy sources increasing, there is opportunity to consider alternative-cropping scenarios to sugar production that may increase financial security and ameliorate environmental impacts caused by conventional management practices. One potential candidate species is napiergrass, a warm-season perennial C4 grass of African origin that produces high biomass yields under tropical conditions. In recent studies, napiergrass produced more than 45 Mg dry wt ha^−1^ year^−1^ in Florida [30] and between 40 and 53 Mg dry wt ha^−1^ year^−1^ in Hawaii [31, 32]. In contrast to sugarcane production in Hawaii, napiergrass is a ratoon-harvested crop that maintains high yields with zero-tillage management and no pre-harvest burning. Regional water shortages from drought and limitations to water access from litigation impose uncertainty on Maui’s agricultural communities. As a result, experimental scenarios include deficit irrigation to better anticipate changes in crop yields and GWP brought on by limited water. For this experiment, the napiergrass plots received the same amount of fertilizer as the sugarcane plots.

For the purpose of creating a C budget for these alternative-cropping scenarios the following assumptions were made: (1) napiergrass fossil-based emissions during the field preparation, harvest, and maintenance operations were considered equal to baseline sugarcane operations due to the similarities in production requirements between the species, (2) seed emissions for napiergrass were calculated based on actual planting rate (seed pieces per plot) relative to the sugarcane seed rate (6500 kg ha^−1^), because a total weight of napiergrass seed was not known, (3) both the lime and herbicide application rate were considered equal to the baseline sugarcane system, regardless of the species or irrigation treatment, (4) deficit irrigation values were adjusted to the known amount of water applied to each of the 100% and 50% treatments, (5) total biomass for each scenario was adjusted based on a 44% reduction in yield found under the deficit irrigation treatments measured in the field at the time, (6) litter decomposition rates and emissions were reduced for the deficit treatments based on residue management and napiergrass decomposition was calculated based on unburned residue N content after harvest, (7) emissions resulting from pre-harvest burn operations were adjusted for the sugarcane 50% scenario by accounting for the reduction in yield under the deficit irrigation, and (8) no SOC accumulates under the baseline scenario due to the incorporation of the pre-burn harvest followed by the associated soil disturbance of conventional tillage operations after harvest.

### Calculating GHG intensity

Greenhouse gas index (GHGI) relates net GWP to crop production by dividing net GWP values by total crop biomass [13]. Net GWP was calculated based on the net C balance for each scenario (described above) and reported in kg C_eq_ ha^−1^ year^−1^. In the first 2 years of the trial, yields at 100% irrigation were 73.9 Mg ha^−1^ year^−1^ for sugarcane and 47.7 Mg ha^−1^ year^−1^ for napiergrass [16]. These values were similar to 2011 sugarcane crop yields data reported from HC&S as 80.4 Mg ha^−1^ year^−1^. Similarly, best-case Hawaiian napiergrass biomass averages reported from Kinoshita et al. [31] and Kinoshita and Zhou [17] were approximately 45 Mg ha^−1^ year^−1^. The measured reductions in yields under the deficit irrigation treatment during the first 2 years of the field trial were 60% for sugarcane (29.4 Mg ha^−1^ year^−1^) and 31% (32.9 Mg ha^−1^ year^−1^) for napiergrass [16]. This metric is useful for comparing scenarios with different yield potentials relative to their impact on GWP and C balance: more positive GHGI values are a stronger source of atmospheric GHGs per unit crop yield, whereas, more negative values are a stronger sink.

## Results and discussion

### Baseline system C balance

**Fig. 1:**
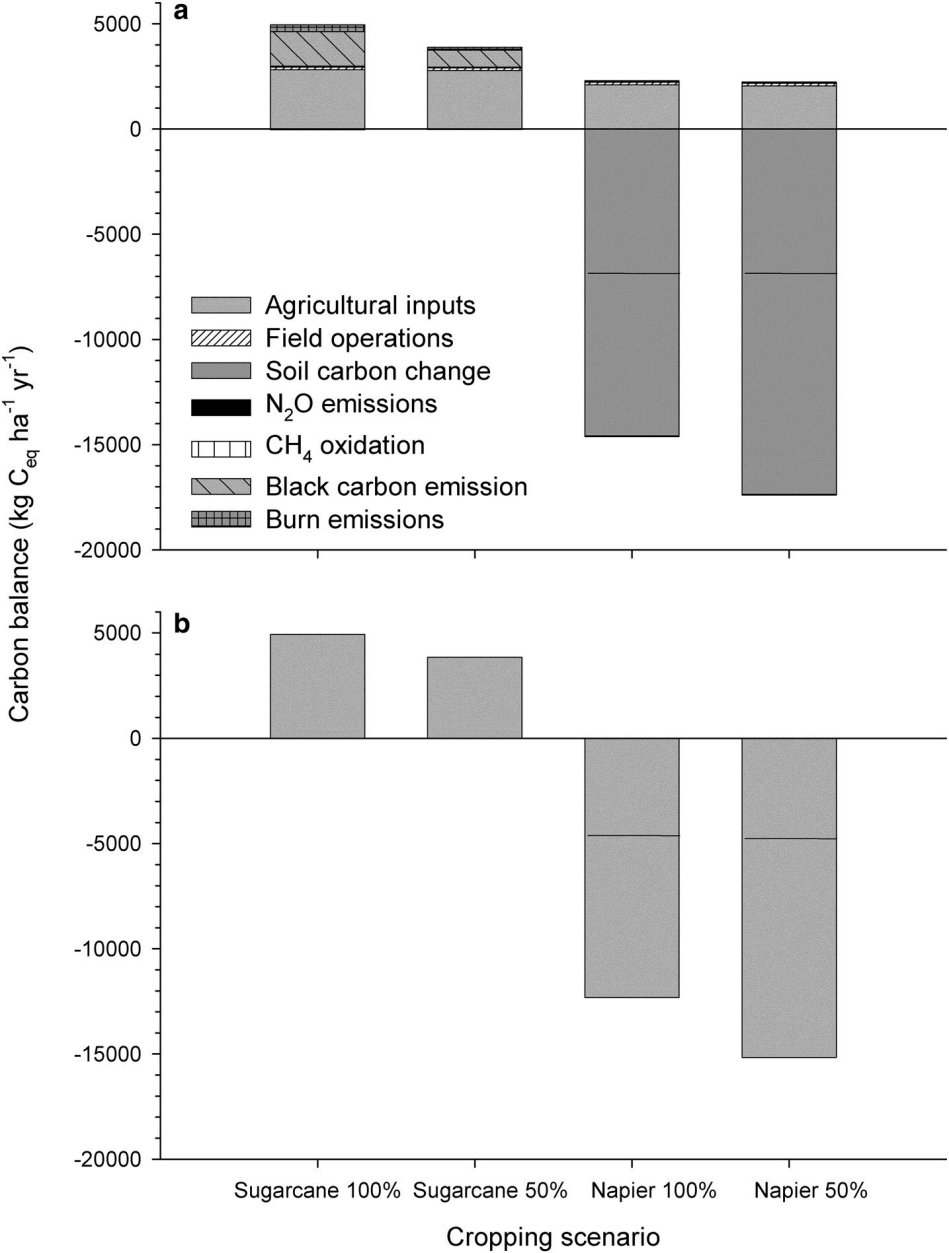
Carbon emission balance by agricultural input and output (**a**), net C balance for each energy crop scenario (**b**)

The majority of C emissions in the baseline scenario (sugarcane 100%) were a result of fossil fuel-based field operation inputs and BC emissions (Fig. 1a). Agricultural inputs together (778 kg C_eq_ ha^−1^ year^−1^) were a smaller contributor to total emissions than field operations (2181 kg C_eq_ ha^−1^ year^−1^) and factors associated with burning (1644 and 320 kg C_eq_ ha^−1^ year^−1^ for BC and CH_4_ + N_2_O emission respectively) (Table 1). Emissions associated with seed propagation comprised 89% of the field operations total input. Individually, the other field operations inputs and soil GHG flux and litter decomposition were minor, contributing less than 100 kg C_eq_ ha^−1^ year^−1^ each). In contrast, 1964 kg C_eq_ ha^−1^ year^−1^ were released through a single burn event. This system was a significant net source of C to the atmosphere, with a net GWP of 4949 kg C_eq_ ha^−1^ year^−1^ (Fig. 1b).

**Table 1:** Agricultural carbon (C) balance of bioenergy cropping scenarios in Maui, Hawaii

C inputs and outputs	Description	kg C_eq_ ha^−1^ year^−1^			
		Sugarcane 100%	Sugarcane 50%	Napiergrass 100%	Napiergrass 50%
Fossil emission					
Field operations					
Field preparation^a^					
Lime application	John Deere 5000–7000 (140–360 HP)	5.2	5.2	5.2	5.2
Subsoiler	John Deere 9000 (540 HP)	12.4	12.4	12.4	12.4
Harrow	John Deere 9000 (540 HP)	3.8	3.8	3.8	3.8
Strip tillage	John Deere 9000 (540 HP)	5.4	5.4	5.4	5.4
Planter	Game (250–300 HP)	5.7	5.7	5.7	5.7
Herbicide application	John Deere 5000–7000 (140–360 HP)	2.1	2.1	2.1	2.1
Harvest^b^					
Cane harvester	John Deere 3522 (337 HP)	63.1	63.1	63.1	63.1
Loader	Cat 950 and John Deere 624 (175–200 HP)	10.8	10.8	10.8	10.8
Hauler	Cat 773B-E (650–675 Hp)	18.1	18.1	18.1	18.1
Fabrication/maintenance^c^					
Farm machinery	Tractors, implements and trucks	45.7	45.7	45.7	45.7
Seed propagation^d^		1950.0	1950.0	1300.2	1300.2
Irrigation^e^		58.7	29.9	58.7	29.9
Field operations subtotal		*2181.2*	*2152.3*	*1531.4*	*1502.5*
Agricultural inputs					
Fertilizer application^f^	344.68 kg ha^−1^ year^−1^ for SC, 243.63 kg ha^−1^ year^−1^ for N	355.0	355.0	236.8	236.8
Herbicide^g^	Applied at a rate 20.307 kg ha^−1^ year^−1^ for first year	114.5	114.5	114.5	114.5
Lime (CaO)^h^	Applied at a rate of 2569 kg ha^−1^ every 2 years	308.3	308.3	308.3	308.3
Agricultural inputs subtotal		*777.8*	*777.8*	*659.6*	*659.6*
Fossil emission subtotal		*2959.0*	*2930.1*	*2190.9*	*2162.1*
Non-fossil emissions					
Litter decomposition^i^		10.3	4.6	41.3	18.2
Pre-harvest burning emissions^j^					
Burn emissions of CH_4_ and N_2_O			320.3	141.0	0.0
Black carbon		1644.0	797.7	0.0	0.0
Soil emisisons^k^					
N_2_O emission		44.5	31.7	62.1	54.0
CH_4_ emission		− 29.4	− 19.7	− 40.9	− 35.4
Non-fossil emissions subtotal		*1989.7*	*955.1*	*62.5*	*36.9*
Total emissions		*4948.6*	*3885.2*	*2253.5*	*2198.9*
Outputs					
Δ Soil carbon (surface layer)^l^		0	0	− 6828	− 6820
Δ Soil carbon (deep profile)^l^		0	0	− 14,571	− 17,359
Net flux (surface layer)				*− 4574.5*	*− 4621.1*
Net flux (deep profile)				*− 12,317.5*	*− 15,160.1*

^a^Data from Lal [20] and Macedo et al. [11] for emissions and EF related to fuel consumption from farming operations during field preparation. Equipment description and HPs gathered from personal communication with HC&S (2013). EF used 0.853

^b^Data from Lal [20] and Macedo et al. [11] for emissions and EF related to fuel consumption from harvest operations. Equipment description and HPs gathered from personal communication with HC&S (2013). EF used 0.853

^c^Values for fabrication and maintenance of equipment from Samson et al. [5]

^d^EF 0.3 used from Six and Jastrow [21], based on total seed weight of 6500 kg ha^−1^ for sugarcane and 4334 kg ha^−1^ for napiergrass

^e^Used Lal [20] conversion factor of kWh = 7.25 × 10^−2^ CE for conversion of total energy used to pump 1 MG of water. Total energy was reduced by 75% based on personal communication with HC&S to account for renewable energy

^f^EF for N fertilization used from Lal [20] and Macedo et al. [11]. EF used 0.97; *SC* sugarcane, *N* napiergrass

^g^Individual herbicide EF were calculated from Lal [20], EF used 5.64

^h^ EF used 0.12 from Macedo et al. [11]

^i^Assuming trash input is 15% of total biomass yield. Based on N content in residual litter of 45 kgN ha^−1^ in unburnt systems and 7 kgN ha^−1^ in burnt systems (Cerri [42]; Galdos et al. [12]; Lisboa et al. [3])

^j^Assuming 15% residue. EF 0.07 kg N_2_O DM burnt and 2.70 kgCH_4_ DM burnt (IPCC 2006; Macedo et al. [11]). BC based on 1.0 kg BC Mg trash burnt and a GWP of 500 relative to CO_2_ (Sanhueza [23])

^k^Used IPCC (2007) GWP of 25 for CH_4_ and 298 for N_2_O

^l^Change (Δ) in SOC was measured by the equivalent soil mass method in year one and two of cultivation, reported value is the mean annual Δ

As of January 2016, HC&S, the last remaining large-scale sugar producer in the Hawaiian islands announced a transition from intensive sugarcane production to diversified agriculture with a commitment to conservation management practices and sustainability. Moving forward, this baseline system may serve as an indicator for improvements in plantation-level sustainability by tracking individual components of fossil and non-fossil fuel based emissions as well as the net GWP. Black C emissions associated with the pre-harvest burn practice already ceased and C costs associated with seed propagation should decline with the phasing-out of 2-year crop rotations. As these components contributed the majority of C emissions to the conventional sugarcane system even under deficit irrigation, the net GWP of the plantation will decrease immediately.

### Comparison of crop scenarios

The differences among crop scenarios for the fossil fuel-based emissions associated with agricultural inputs and field operations were small compared to the differences associated with pre-harvest burn emissions and soil C stock under ratoon harvest and zero-tillage management (Fig. 1a). As a result, the net GWP was greatest for the sugarcane 100% followed by sugarcane 50% (Fig. 1b). The deficit irrigation treatment in sugarcane resulted in a 21.5% reduction in the net GWP to 3885 kg C_eq_ ha^−1^ year^−1^ (Table 1). Largely because the napiergrass scenarios do not include burning and accumulate soil C, the net GWP was negative (and therefore a sink rather than a source of C) for both the 100% and 50% scenarios.

The critical component of the net C sink under alternative scenarios was the cultivation of a ratoon-harvested energy crop that maintains high yields with zero-tillage management and requires no pre-harvest burning. In this study, we focused on the cultivation of napiergrass; however, other grasses such as sugarcane and energycane have the same potential to improve GWP from the conventional sugarcane practices. Recent studies suggest a range of soil C responses from perennial grasses depending on genotype [33], species or accession [32], and soil properties [34]. The soil C change from the initial shift to ratoon harvested napiergrass from over 100 years of intensive, monocropping cultivation was large and rapid. The annual soil C change is likely to decrease over time as the initial phase of accumulation ends and C saturation approaches [35]. How high soil C stock will go and how long the system will take to reach equilibrium remains unknown.

The difference between burned and un-burned GHG balances has been well documented in recent literature. For example, Lisboa et al. [3] found that over 17% of total emissions from sugarcane production in Brazil was due to pre-harvest trash burning and Galdos et al. [12] found that the single largest source of emissions in a traditional sugarcane system in Brazil was also due to biomass burning, resulting in over 1732 kg C_eq_ ha^−1^ year^−1^. Our values ranged from 938 to 1964 kg C_eq_ ha^−1^ year^−1^ depending on the irrigation treatment.

The sugarcane scenarios generated the largest fossil-based emissions: 2959 and 2930 kg C_eq_ ha^−1^ year^−1^ for the 100% and 50% respectively, whereas the 100% and 50% napiergrass scenarios were less: 2191 and 2162 kg C_eq_ ha^−1^ year^−1^ respectively (Table 1). The single largest contributor to fossil emissions for all scenarios was the production of seed: 1950 and 1300 kg C_eq_ ha^−1^ year^−1^ for sugarcane and napiergrass respectively, which accounted for more than 60% of fossil-based emissions from all of the scenarios. The seed emissions were the driving factor for the differences in fossil based emissions between the sugarcane and napiergrass scenarios. Napiergrass may be ratooned for up to 10 years without yield declines [31], so seed propagation should decline depending on management decisions concerning kill-harvest cycles.

Fertilizer contribution to GWP was over 12% of total fossil emissions for sugarcane and 11% for napiergrass, regardless of the irrigation scenario. The application of mineral fertilizers contributes a substantial amount of emissions in other sugarcane systems [3, 11]. The potential exists for the fertilizer contribution to GWP to increase over time. Many of the large inputs accounted for in this study (seed propagation, liming and field preparation) are associated with the first-year of cultivation. Their effects on overall emissions are expected to decline, especially in subsequent years that allow for ratoon harvests. Therefore, the effects of N fertilizer may become more important because it will most likely remain a constant source of inputs as nutrients are added to the system each year in order to maintain yields [6]. However, in the napiergrass scenarios, as soil quality improves over time with the accumulation of organic matter under conservation practices, the fertilizer requirement may be reduced [36].

Although the reduction in irrigation did not have a large direct effect on the fossil fuel based emissions, the impact on biomass yield and soil C in deep soil horizons in napiergrass was high. Many of the inputs for GHG accounting require biomass yields in order to calculate the associated emissions. Our estimates of the yield reduction caused by the 50% deficit resulted in a substantial reduction in litter decomposition and burn-related emissions. However, these reductions only influenced the net C balance of the sugarcane scenarios. The difference in net C balance between the napiergrass irrigation scenarios were minimal if only the surface soil horizon was considered, but substantial if the deep soil profile was considered.

Driven by a lack of pre-harvest burn, non-fossil based emissions were markedly different between the sugarcane and napiergrass systems (Fig. 1a). Even within the sugarcane scenarios, the 100% irrigation scenario produced nearly double the emissions as the 50% scenario (Table 1) due to reduction in biomass under the deficit irrigation. Although a minor contributor, litter decomposition was reduced under deficit irrigation due to lower yield, and therefore lower residual plant litter. Soil N_2_O and CH_4_ fluxes were comparable between the scenarios [16].

Soil C increased rapidly from the baseline during the 2-year crop cycle [16] resulting in substantial C credit towards the net GWP for the napiergrass scenarios (Fig. 1a). In the surface layer, the annual increase in soil C was nearly identical between the napiergrass scenarios (Table 1). However, soil C increased more in the deficit irrigation scenario if the deeper soil horizons also were considered. Because of the large contribution of soil C output to GWP in the napiergrass scenarios, the net GWPs were − 4574 and − 4621 C_eq_ ha^−1^ year^−1^ for the napiergrass 100% and 50% scenarios respectively if only accounting for the surface soil layer. If accounting for the deep soil profile, then the net GWP decreases further to − 12,317 C_eq_ ha^−1^ year^−1^ (napiergrass 100%) and − 15,160 C_eq_ ha^−1^ year^−1^ (napiergrass 50%).

The vast majority of studies take only the surface soil layer (typically 0–15 or 30 cm) into account for C stock assessment and inventory [37]; however, this practice ignores deeper soil processes associated with roots, the rhizosphere and dissolved organic matter transport. As pointed out above, these processes could result in soil priming (and C loss) [38], soil C accrual etc. As a tropical, perennial C4 grass, napiergrass is known to generate deep, extensive root systems in search of nutrients and water, particularly during drought [39]. Accordingly, soil C accrual as a result of root-derived inputs to the soil was even more prominent in deeper horizons of the deficit irrigation treatment compared to the 100% of plantation practice treatment.

### Greenhouse gas intensity

Per unit of biomass yield, the most GHG intensive scenario was sugarcane 50% (GHGI = 0.11) and the least intensive was napiergrass 50% when the deep soil profile was considered in the net GWP calculation (GHGI = − 0.77) (Table 2). The reduction in sugarcane yield with 50% less water was not matched proportionately by reductions in C emissions, so the deficit irrigation did not provide a net benefit to the GWP of the system. For napiergrass, the opposite was true − 50% less water improved the GHGI index (i.e., made it more negative), particularly if the C accrual in the deep soil profile was considered. Even if only the surface layer of soil C accrual was accounted for, because the net C sink was not different and the yield was reduced, the deficit irrigation provided a net benefit to the GWP of the system.

**Table 2:** Greenhouse gas index (GHGI) of the baseline and three alternative cropping scenarios

	Sugarcane 100%	Sugarcane 50%	Napiergrass^a^ 100%	Napiergrass^a^ 50%
Surface layer	0.06	0.11	− 0.10	− 0.23
Deep profile	0.06	0.11	− 0.27	− 0.77

^a^For napiergrass, GHGI were calculated from net C balances that included both surface layer and deep profile soil C accrual

## Conclusions

Moving away from intensive sugar production, the napiergrass systems demonstrated the greatest potential for climate change mitigation, whereas sugarcane scenarios that maintain conventional harvest practices are large sources of C to the atmosphere. Reduced emissions from sugarcane systems will rely on removal of burn related emissions and through the reduction of fertilizer use or improvement of nutrient use efficiency in these systems [40]. In the Pacific Basin, future scenarios for energy or fuel production on former sugarcane land should concentrate on ratoon-harvested crops that maintain yields under zero-tillage management for long intervals between kill harvest (if they are required at all). For napiergrass on Maui, deficit irrigation from the conventional sugarcane practice provided the greatest net climate change mitigation and reduced water usage should be an important aspect of planning a sustainable, diversified agricultural landscape under pressure for improved water resource management.

## Abbreviations

BC: black carbon
C: carbon
C_eq_: carbon equivalent
CH_4_: methane
CO_2_: carbon dioxide
EF: emission factor
EISA: Energy Independence and Security Act
GHG: greenhouse gas
GHGI: greenhouse gas index
GWP: global warming potential
HCEI: Hawaii Clean Energy Initiative
HC&S: Hawaiian Commercial & Sugar
N_2_O: nitrous oxide
